# Prevalence and Characterisation of *Clostridium perfringens* Isolates in Food-Producing Animals in Romania

**DOI:** 10.3390/microorganisms11061373

**Published:** 2023-05-24

**Authors:** Corina Beres, Liora Colobatiu, Alexandra Tabaran, Romolica Mihaiu, Marian Mihaiu

**Affiliations:** 1 Department of Animal Breeding and Food Science, Faculty of Veterinary Medicine, University of Agricultural Sciences and Veterinary Medicine, Manastur Street No. 3/5, 400372 Cluj-Napoca, Romania; corinaberes@yahoo.com (C.B.); alexandra.lapusan@usamvcluj.ro (A.T.); marian.mihaiu@usamvcluj.ro (M.M.); 2 Department of Medical Devices, Faculty of Pharmacy, Iuliu Hatieganu University of Medicine and Pharmacy, Victor Babes Street No. 8, 400012 Cluj-Napoca, Romania; 3 Department of Management, Faculty of Economic Sciences and Business Administration, Babes Bolyai University, Mihail Kogalniceanu Street No.1, 400084 Cluj-Napoca, Romania; romolica.mihaiu@econ.ubbcluj.ro

**Keywords:** *Clostridium perfringens*, gastroenteritis, prevalence, toxins, antimicrobial resistance, food-producing animals

## Abstract

The aim of the current study was to investigate the prevalence of *Clostridium perfringens* (*C. perfringens*) recovered from animal faeces, as well as to determine the antimicrobial susceptibility of such isolates. A total of 14 (14/100; 14%) *C. perfringens* isolates were isolated from the 100 analysed samples (twelve recovered from faecal samples collected from pigs and two from veal calves’ faecal samples). The preponderant genotype was type A, with all isolates being *cpa*-positive. The most potent antimicrobial agents against *C. perfringens* proved to be vancomycin, rifampicin and lincomycin. A strong resistance to tetracycline (71.4%), penicillin (64.2%), erythromycin (42.8%) and enrofloxacin (35.7%) was also observed. To the best of our knowledge, this is the first analysis regarding the prevalence, characterization and antimicrobial susceptibility of *C. perfringens* in food-producing animals in Romania, adding further evidence for the probable role of animals as a source of resistant *C. perfringens* strains.

## 1. Introduction

*Clostridium perfringens* (*C. perfringens*) is a ubiquitous, anaerobic, Gram-positive, spore-forming bacteria, extensively distributed in the environment, ranking amongst the most important pathogens living in the intestinal tracts of both humans and animals [1]. It causes a wide range of histotoxic and gastrointestinal infections, most of these being mediated by one or more toxins [2,3,4,5]. Based on the ability to produce six major toxins, namely alpha (α), beta (β), epsilon (ε), iota (ι), enterotoxin (CPE) and NetB, *C. perfringens* isolates are usually classified into seven different toxin types/biotypes (from A to G), with each distinct toxin type being generally associated with certain diseases [6,7]. The alpha toxin, encoded by the *cpa* gene, and located on the chromosome, is considered to be produced by all toxigenic types of *C. perfringens*. Type B produces the β and ε toxin, type C only produces the β toxin, type D the ε toxin, type E the ι toxin, while type G produces the NetB [8]. Type F strains are known to produce the enterotoxin (encoded by *cpe*), the main virulence factor implicated in foodborne gastroenteritis outbreaks, and have been previously known as *cpe*-positive *C. perfringens* type A strains [3,9]. Moreover, type F strains have been exclusively linked to food poisoning mediated by *C. perfringens* [10]. 

Each particular toxin type is considered to be associated with specific diseases. For instance, type A strains can cause enterocolitis in pigs and horses or necrotic enteritis in chicken, but are also responsible for gas gangrene in humans, while type B is involved in necrotizing enteritis in sheep and cattle. Type C strains are known to cause necrohaemorrhagic enteritis in piglets, horses or sheep and type D has been mainly associated with enterotoxaemia in goat, sheep or cattle. Type E strains also cause enteritis in different animal species, such as sheep or cattle. As previously mentioned, type F is considered to be linked mostly to food poisoning in humans and animals [3,6,10]. Therefore, the determination of the toxin type of various *C. perfringens* isolates is considered to be a critical step in properly evaluating the risk associated with such strains. 

Different antimicrobial agents such as tetracycline, chloramphenicol, lincomycin, bacitracin, ampicillin, metronidazole and even imipenem have been used over the years in the prophylaxis and treatment of infections caused by *C. perfringens* [3,10,11]. Unfortunately, the long-term, as well as frequent, use of such agents has facilitated the emergence of antimicrobial resistance, compromising the efficacy of the available antimicrobial agents administered in the treatment of *C. perfringens* infections [12]. Several studies have indicated that, as a result, the antimicrobial resistance to specific drugs such as tetracycline, erythromycin, lincomycin or chloramphenicol has significantly increased in recent years, causing important difficulties in regard to clinical treatment as well [12]. Pathogens which are resistant to antimicrobials that are considered to be critically important in the treatment of human disease are associated with particularly important consequences [13].

Several antimicrobial resistance genes have also been identified in *C. perfringens* isolates originating from animals, which are known to play a major role in the transfer of resistance to other bacteria [14]. Genes encoding antimicrobial resistance determinants (ARD), carried by resistant bacteria, may be transferred to other bacteria of clinical significance, with the rapid development and dissemination of antimicrobial resistant bacteria resulting not only from intense antimicrobial selective pressure, but also from the exchange of ARD among the gut microbiota population. To date, various resistance genes have been identified in *C. perfringens* isolates, including *tetA*(P), *tetB*(P), *tet*(K), *tet*(L) (conferring resistance to tetracycline), *erm*(B), *erm*(Q) (macrolide resistance genes), *lnu*(A), *lnu*(B), *lnu*(P) (responsible for lincomycin resistance), *catP* and *catQ* (conferring resistance to chloramphenicol) and even *optrA*, as the first identification of oxazolidinone/phenicol resistance in *C. perfringens* isolates. 

Despite being considered as one of the most common causes of foodborne illness worldwide, there is still much to be learned about the characteristics of *C. perfringens*, particularly in terms of prevalence and distribution in various environments. 

Information about *C. perfringens* in the food supply chain in Romania is lacking. There are almost no data recorded about the prevalence, transfer and antimicrobial resistance of *C. perfringens* strains of animal origin and food of animal origin in our country. 

Therefore, the current study was conducted in order to investigate the prevalence and toxin type of *C. perfringens* isolated from animal faeces, as well as to determine the antimicrobial susceptibility of the obtained isolates.

## 2. Materials and Methods

### 2.1. Sampling

A total of 100 samples of animal faeces, 60 from pigs and 40 from veal calves (as enterotoxaemia due to *C. perfringens* is more likely to affect calves), were collected from July to September 2022, from two geographically distinct farms located in the centre of Romania (46°44′02.23″ N, 23°53′59.11″ E and 45°54′42.80″ N, 23°20′36.57″ E, respectively). All the samples (approximately 50 g) were collected aseptically, using sterile cotton swabs, directly from the rectum, and then transported to the laboratory in ambient conditions, stored at 4 °C and processed within 24 h.

### 2.2. C. perfringens Isolation

Robertson cooked meat (RCM; Merck, Darmstadt, Germany) medium was used as an enrichment broth for the isolation of the target strains. The faecal samples were inoculated in the RCM medium and then incubated at 37 °C for 24 h, under anaerobic conditions (CO_2_GEN; Oxoid Ltd., Basingstoke, Hampshire, UK). The enrichment culture obtained was cultured onto tryptose sulfite cycloserine agar (TSC; Oxoid Ltd., Basingstoke, Hampshire, UK) and incubated at 37 °C, for a further 24 h (CO_2_GEN; Oxoid Ltd., Basingstoke, Hampshire, UK). The identification of *C. perfringens* isolates was achieved by observing the formation of specific black colonies, with a white halo and microscopic morphology. Vitek^®^ 2 ANC cards for anaerobes (intended for use with the Vitek 2 system) (bioMérieux, Marcy l’Etoile, France) were further used for the biochemical confirmation of the strains. 

### 2.3. Toxinotyping of the C. perfrigens Isolates

The genomic DNA was extracted with the help of the QIAamp DNA Micro Kit (Qiagen), according to the manufacturer’s instructions. Multiplex PCR was performed in order to detect the presence of four toxin genes, namely *cpa*, *cpe*, *becA* and *becB*, as previously reported [9]. Briefly, PCR was conducted in a final volume of 25 μL reaction mixture, comprising the following: 12.5 μL × 2 Multiplex PCR Master Mix (Qiagen), 0.4 μM primers for the *cpa* and *cpb* genes, 0.2 μM primers for *becA* and *becB* genes and 1 μL of template solution. PCR was performed under the following conditions: denaturation at 95 °C for 5 min, 30 cycles of denaturation at 95 °C for 30 s and a final extension step at 68 °C for 10 min. The PCR products were analysed by electrophoresis on 3% agarose gels. The sequences of the four pairs of primers used in order to amplify the above-mentioned genes are presented in Table 1. The DNA of *C. perfringens* type A isolate, which was already available in the laboratory, was used as a positive control during the PCR reactions, while reference strain *Listeria monocytogenes* ATCC 51776 was used as a negative control. The presence of the specific PCR products (324 bp for *cpa*, 233 bp for *cpe*, 400 bp for *becA* and 416 bp for *becB*, respectively) confirmed the possession of each mentioned gene.

### 2.4. Antimicrobial Susceptibility Testing

Susceptibility to tetracyclines (tetracycline, 30 μg), macrolides (erythromycin, 15 μg), aminoglycosides (gentamicin, 10 μg), glycopeptides (vancomycin, 10 μg), rifamycins (rifampicin, 5 μg), β-lactams (penicillin, 10 units), polypeptides (bacitracin, 10 units), lincosamides (lincomycin, 2 μg) and fluoroquinolones (enrofloxacin, 5 μg) was determined by the disk diffusion method, on Mueller–Hinton agar (Merck, Darmstadt, Germany), according to the guidelines provided by the Clinical and Laboratory Standards Institute (CLSI) [18]. The agar plates were inoculated with cultures of *C. perfringens*, followed by the application of an antimicrobial paper disk. The plates were afterwards incubated at 37 °C, under anaerobic conditions, for 24 h. The diameters of the zone of inhibition around the disk were measured with the help of a digital calliper, with resistance being defined as follows: tetracycline < 24 mm; erythromycin < 22 mm; gentamicin < 19 mm; vancomycin < 17 mm; rifampicin < 26 mm; penicillin < 26 mm; bacitracin < 22 mm; lincomycin < 17 mm; enrofloxacin < 22 mm. *Staphylococcus aureus* ATCC 29213 was used as the reference strain. The antimicrobial agents used in order to carry out the antimicrobial susceptibility testing were selected based on their use in veterinary medicine, as well as on resistance profiles of *C. perfringens* isolates, as reported in previous studies [3,19].

## 3. Results

### 3.1. Prevalence of C. perfringens

A total of 14 *C. perfringens* isolates were recovered from the 100 analysed samples, with 12 isolates being detected in the faecal samples collected from pigs (12/60; 20%) and only 2 isolates being recovered from the veal calves’ faecal samples (2/40; 5%).

### 3.2. Toxinotyping of the C. perfringens Isolates

**Figure 1:**
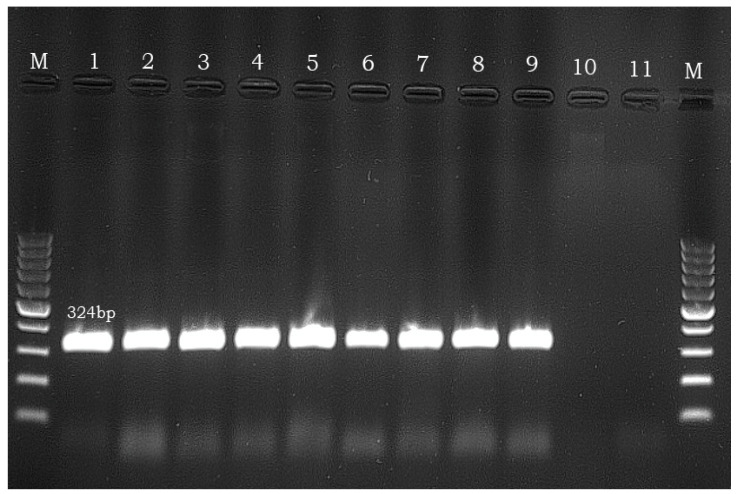
Representative agarose gel run showing the PCR detection of the *cpa* gene. M: molecular weight marker; Lane 1: positive control (*Clostridium perfringens* type A-laboratory collection); Lanes 2–9: *Clostridium perfringens* isolates; Lane 10: negative result; Lane 11: negative control (*Listeria monocytogenes* strain ATCC 51776).

Toxin typing of the recovered 14 *C. perfringens* isolates revealed that all of these were type A, being positive only for the *cpa* gene, and negative for the *cpe*, *becA* and *becB* genes (Figure 1).

The susceptibility profiles of the *C. perfrimgens* isolates are presented in Table 2.

The results indicate that all recovered *C. perfringens* isolates are susceptible to vancomycin, rifampicin and lincomycin. Resistance to tetracycline (71.4%), penicillin (64.2%), erythromycin (42.8%) and enrofloxacin (35.7%) was also observed. Nevertheless, only one *C. perfringens* isolate proved to be multidrug resistant, showing resistance to at least three different classes of antibiotics, namely tetracyclines (tetracycline), β-lactams (penicillin) and fluoroquinolones (enrofloxacin). 

## 4. Discussion

The infection caused by *C. perfringens* has usually been associated with a wide range of histotoxic and gastrointestinal diseases in both humans and animals [20,21]. It is also considered to be one of the most prevalent foodborne pathogens [22]. Studies regarding the prevalence and antimicrobial resistance of *C. perfrigens* isolates performed so far have been mostly focused on humans, as well as food-producing animals including swine, chicken, turkey, cattle, but also sheep, goat, or dromedary camels [4,10,21,23,24,25]. 

To the best of our knowledge, in Romania, at the moment, there are almost no available data regarding the prevalence of *C. perfringens* isolated from food animals. 

Overall, a total of 14 (14%) *C. perfringens* isolates were recovered from the 100 analysed samples, with the majority of these being detected in the faecal samples collected from pigs (12 isolates). Different studies have analysed the prevalence and distribution of *C. perfringens* in various countries, including India, South Korea, the United States, China, Thailand, Canada, Egypt, Turkey, Sweden, Norway, Denmark, Spain or Belgium [4,11,19,24,26,27,28,29,30,31,32,33]. The prevalence of *C. perfringens* identified in our study appears to be lower compared with previous studies performed in other countries, which reported a prevalence ranging from 10% to 72% [10,19,25,30,33,34,35,36].

For example, a study performed by Li et al. in China, which also included faecal samples collected from pigs and chickens, reported a high isolation rate of *C. perfringens* in pigs (72%), as well as in chickens (23.4%) [10]. However, another study which focused on identifying the prevalence of *C. perfringens* among different animal species in India reported a much lower overall prevalence of 10.76% compared to other studies performed in the same country [19]. Several other previous studies showed variable prevalence rates, such as 12.6% in Spain, 30% in the United States, 31.7% in Turkey, 41.7% in Egypt, 62% in Canada and 64% in the Czech Republic [4,31,33,37,38].

The samples were collected during summer (July to the beginning of September), which might explain the lower-than-expected recovery of *C. perfringens* isolates due to the warm and dry season. It has been previously reported that infections with *C. perfringens* are more frequent during the cold season, as well as in early winter [39]. 

The isolation levels of *C. perfringens*, which have been mentioned so far, might seem quite contrasting, although several studies showed that the incidence of *C. perfringens* in food-producing animals varies widely from one country to another. It is considered that such differences might be due to the use of different detection methods, and it has also been postulated that even if *C. perfringens* mainly colonizes the intestine, its prevalence rate is subject to the living environment of the host animal [29].

Moreover, *C. perfringens* isolates have also been frequently recovered from meat samples of different origins, such as chicken, beef or pork [3]. 

All the *C. perfringens* isolates recovered in the present investigation were identified as type A (*cpa*-positive), which is in accordance with earlier reports regarding the global dominance of type A, especially in pigs [19,25,37]. *C. perfringens* type A is considered to be the most common among the toxigenic types implicated in foodborne poisoning in the United States, Europe and Japan [40]. Moreover, all *C. perfringens* isolates proved to be *cpe*-negative. Other previous studies have also identified only *cpa*-positive *C. perfringens* isolates of different origins, suggesting that *cpa* might be a universal gene, specifically in *C. perfringens* isolated from meat samples [8,32,41,42]. Wen et al. also reported that all *C. perfringens* isolates from beef, chicken and pork meats recovered in their study were classified as type A. Moreover, another survey performed in Belgium found that 71 *C. perfringens* isolates from chicken meat were identified as type A, while Aras et al. also reported a high percentage of type A (*cpa*-positive) *C. perfringens* isolates from meat samples in Turkey [4]. The *cpa* gene is usually located on the chromosome, while the *cpe* gene may be located either on the chromosome or on the plasmids [6]. The *cpe* gene encodes the enterotoxin. However, only a low percentage of the *C. perfringens* isolates were proven to possess such a gene (less than 5%). Moreover, it has been previously reported that the loss or acquisition of plasmids might be responsible for the changes in toxin type observed in certain strains [8]. Jang et al., who also identified only *cpa*-positive/*cpe*-negative *C. perfringens* isolates, considered that such strains might have lost their mobile components, which could explain why only the *cpa* gene was identified in the *C. perfringens* strains [3]. 

When it comes to the antimicrobial susceptibility profiles of the isolated strains, the results were judged based on the inhibition zone diameters of *Staphylococcus aureus* (as there are no *C. perfringens* breakpoints available from CLSI). All *C. perfringens* isolates recovered in our study proved to be susceptible to vancomycin, rifampicin and lincomycin, which is consistent with other reported results [10,19]. A very low resistance to vancomycin has also been identified by Li et al. in faecal samples collected from chickens and pigs [10]. Such an outcome might be related to the negligible use of these particular antimicrobial agents in the treatment of *C. perfringens* infections in food-producing animals. However, higher lincomycin resistance rates have been reported by other authors [19,43]. 

A large proportion of the isolates were resistant to tetracycline (71.4%), penicillin (64.2%), erythromycin (42.8%) and enrofloxacin (35.7%). Antimicrobial resistance to these drugs (especially to tetracycline and erythromycin) has been previously highlighted in the case of other important foodborne pathogens isolated in Romania, including *Salmonella* spp., *Escherichia coli* or *Campylobacter* spp. [44]. Tetracycline resistance seems to be common among animal isolates of *C. perfringens* and has been well documented [10,19,25,26,28]. In a study performed by Anju et al., tetracycline resistance was found to be 40% among the recovered isolates. Similarly to our study, the same authors have also reported a low resistance to lincomycin, which was only identified in chicken and turkey isolates. This particular finding was considered to be related to the use of this antimicrobial substance in the treatment of necrotic enteritis in poultry [19]. Jang et al. have even shown that all *C. perfringens* isolates recovered from meat samples in their study were 100% resistant to tetracycline. Antimicrobials have long been used in livestock, either as growth promoters in certain countries, or to treat different infections. Antimicrobial drugs such as tetracycline are still used to control *C. perfringens* in many countries, including Brazil, Belgium, Denmark and Switzerland [41]. It is a well-known fact that the improper use of antimicrobials in animals influences and increases the bacterial resistance to such substances, even leading to the emergence of multidrug-resistant bacteria, as well as residues of antimicrobials in the environment. Moreover, bacteria are also capable of transferring resistant genes to each other, thus subsequently causing and promoting multidrug resistance. Resistance to tetracycline is attributed to many resistant genes, such as *tetA*(P), *tet*B(P), *tet*(M), *tet*(L) and *tet*(K) and these have been frequently identified in *C. perfringens* isolates [19]. 

The results reported in the current study, as well as the ones reported in several previous studies, reveal that the antimicrobial resistance profiles of *C. perfringens* may vary significantly between different countries. 

In our study, the disk diffusion method was used in order to analyse the antimicrobial susceptibility of the recovered *C. perfringens* isolates. The method is frequently employed in such determinations due to its ease of use, although some authors consider that it is not as accurate for anaerobic organisms as it is for aerobic ones. Therefore, this particular aspect, as well as the sample size used in this study, which may be considered as low, might be considered as limitations of our work. 

## 5. Conclusions

In conclusion, the study investigated the prevalence, toxin type, as well as the antimicrobial susceptibility of *C. perfringens* isolates recovered from faecal samples collected from pigs and veal calves. *C. perfringens* were detected in the majority of the pig faecal samples. All isolates were identified as type A and *cpa*-positive. The *C. perfringens* isolates recovered in the current study proved to be highly resistant to tetracycline, penicillin and erythromycin, but susceptible to vancomycin, rifampicin and lincomycin. 

To the best of our knowledge, this is the first study reporting the prevalence and antimicrobial resistance of *C. perfringens* in food-producing animals in Romania.

Among the limitations of the study, we could mention the sample size which was relatively small. However, the study provides a baseline for the future surveillance and characterization of *C. perfringens* in food-producing animals, food and the environment in Romania. The results provide useful insight for veterinarians, as well as public health specialists implicated in the management of *C. perfringens* infections, and highlight the need for continued efforts to prevent and control the spread of this bacterium in the food supply. 

## Figures and Tables

**Table 1:** Primers used to detect the presence of *cpa*, *cpe*, *becA* and *becB* toxin genes.

Gene	Name of Primer	Primer Sequence (5′–3′)	Product Size (bp)	Reference
*cpa*	CPA-F	gctaatgttactgccgttga	324	[15]
	CPA-R	cctctgatacatcgtgtaag		
*cpe*	CPE-F	ggagatggttggatattagg	233	[9,15,16]
	CPE-R	ggaccagcagttgtagata		
*becA*	*becA*-F	caatggggcgaagaaaatta	499	[17]
	*becA*-R	aaccatgatcaattaaaacctca		
*becB*	*becB*-F	tgcaaatgacccttacactga	416	[17]
	*becB*-R	agattggagcagagccagaa		

**Table 2:** Antimicrobial susceptibility of the *C. perfringens* isolates.

Antimicrobial Agent	Disc Concentration	Number of Resistant Isolates	Resistance (%)
Tetracycline	30 μg	10	71.4
Erythromycin	15 μg	6	42.8
Gentamicin	10 μg	1	7.1
Vancomycin	10 μg	0	0
Rifampicin	5 μg	0	0
Penicillin	10 UI	9	64.2
Bacitracin	10 UI	2	14.2
Lincomycin	2 μg	0	0
Enrofloxacin	5 μg	5	35.7

## Data Availability

Not applicable.
